# Process Optimization and Quality Components Analysis of γ-Aminobutyric Acid Pickled Tea

**DOI:** 10.3390/foods13142287

**Published:** 2024-07-20

**Authors:** Qiuyu Luo, Qinmei Li, Jiayu Li, Wei Xu, Ling Lin

**Affiliations:** College of Horticulture, Sichuan Agricultural University, Chengdu 611130, China; 202001479@stu.sicau.edu.cn (Q.L.); 202101569@stu.sicau.edu.cn (Q.L.); 202101585@stu.sicau.edu.cn (J.L.); xuweianti@sicau.edu.cn (W.X.)

**Keywords:** pickled tea, γ-aminobutyric acid, anaerobic fermentation, process optimization, biochemical components

## Abstract

Pickled tea is an anaerobically fermented tea common in Thailand, Myanmar and Yunnan minority areas. γ-aminobutyric acid (GABA) is non-protein amino acid with multiple bioactives, which can be easily produced under anaerobic conditions. During the processing of pickled tea, controlling the process parameters is effective for the production of GABA-rich products; however, the precise parameters remain to be clarified. In the present study, the fresh leaves of *Camellia sinensis* (L.) Kuntze (*C. sinensis*) ‘FudingDabai’, *C. sinensis* ‘MabianLv No. 1’, *C. sinensis* ‘Wuniuzao’ and *C. sinensis* ‘Fuxuan No. 9’ were used as raw materials to process GABA-rich pickled tea. Single-factor and orthogonal experiments were conducted to determine the best tea cultivars and optimize the best processing parameters via comparing the content of GABA, tea polyphenols (TPs) and other biochemical components of GABA-rich pickled tea. The results of the signal-factor experiment showed that the fresh leaves of *C. sinensis* ‘MabianLv No. 1’ had the highest GABA content of 2.61 mg·g^−1^ after treatment with vacuum for 6 h; therefore, *C. sinensis* ‘MabianLv No. 1’ was selected as the raw material for the subsequent experiments. Orthogonal experiments showed that the highest GABA content of 2.53 mg·g^−1^ was found in the pickled tea with 8 h of vacuum treatment, 20 min of rolling after microwave fixing, 20 min of spreading and 20 d of anaerobic fermentation at room temperature. Further, the sensory evaluation showed that it possesses a strong sour taste with a slight sweetness and a light yellow color and better comprehensive quality. This indicates that these parameters are optimal for the processing of GABA-rich pickled tea. This study will provide scientific basis for the subsequent production of high GABA tea.

## 1. Introduction

Pickled tea is an anaerobically fermented tea with a special flavor, which is made from the fresh leaves of tea (*Camellia sinensis* (L.) Kuntze) by natural fermentation and is popular among the ethnic minorities of Bulang and Deang in Yunnan Province. The processing of pickled tea includes fixing and fermentation; specifically, fresh leaves treated with steam or boiling water are packed into bamboo tubes and buried underground for natural fermentation, and then the pickled tea is obtained [1]. Similar products include Japanese Goshichya, Awabancha, Myanmar’s Teh Tarik and Thailand’s Miang, etc. [2]. Due to the great market potential of specialty tea products, as well as the importance of national cultural heritage and protection, studies on pickled tea have attracted highly attention [3].

γ-Aminobutyric acid (GABA), a four-carbon non-protein amino acid, is widely distributed in vertebrates, plants and microorganisms [4]. It is an inhibitory neurotransmitter with a variety of bioactivities [5], such as reducing neuronal activity, lowering blood pressure and preventing atherosclerosis [6]. Furthermore, it is effective in several psychiatric disorders like epilepsy and Parkinson’s disease [4,7,8]. However, GABA decreases with the increase of stress and age, rendering the dietary supplementation of GABA crucial [9]. The content of GABA in plants is extremely low, usually ranging from 0.3 to 32.5 μmol·g^−1^ [10]. It has been reported that the enrichment of GABA in plants is stress-related, and GABA accumulates rapidly when plants are subjected to hypoxia, cold shock and salt stress [11,12,13,14,15]. This suggests that GABA-enriched functional foods can be obtained via stress treatment or microbial fermentation, which has been a focus of food nutrition [16].

Currently, numerous studies have investigated the fermentation of pickled tea and the development of tea products with high GABA content. However, most of these studies have concentrated on the identification of dominant microorganisms during fermentation and the effects of anaerobic treatment on the quality of tea leaves, and fewer studies have focused on the production of GABA-rich pickled tea via process optimization in the anaerobic fermentation of pickled tea. Furthermore, the constituents [17] as well as the GABA content of pickled tea made from different varieties vary.

Therefore, in the present study, the fresh leaves of one bud with two or three leaves of four tea cultivars (*C. sinensis* ‘FudingDabai’, *C. sinensis* ‘MabianLv No. 1’, *C. sinensis* ‘Wuniuzao’ and *C. sinensis* ‘Fuxuan No. 9’) in summer were treated first by vacuum, and *C. sinensis* ‘Mabianlv No. 1’ was screened as the raw material for process optimization experiments by comparing the GABA content. Then, the parameters of the GABA-rich pickled tea production process were optimized by single-factor and orthogonal tests, respectively. The findings can provide a reference for the comprehensive utilization of summer and autumn tea resources, the improvement of pickled tea quality and the research and development of functional tea foods.

## 2. Materials and Methods

### 2.1. Materials and Reagents

Fresh leaves (one bud with two or three leaves) of *C. sinensis* ‘FudingDabai’, *C. sinensis* ‘MabianLv No. 1’, *C. sinensis* ‘Wuniuzao’ and *C. sinensis* ‘Fuxuan No. 9’ were plucked in the Mabian Yi Autonomous County, Sichuan Province, on 25 July 2023. These tea cultivars are national (China) or provincial asexual varieties and possess unique registration numbers. Methyl isothiocyanate and trimethylamine (Chromatographic grade) were purchased from Ling En Technology Co., Ltd. (Shanghai, China). γ-Aminobutyric acid (chromatographically pure) and catechin standards (≥98%) were obtained from Yuanye Bio-technology Co., Ltd. (Shanghai, China). Folinol was from Solaibao (Beijing, China). L-glutamic acid, stannous chloride dehydrate, methanol and acetonitrile were purchased from Chengdu Colon Chemical Co., Ltd. (Chengdu, China).

### 2.2. Vacuum Treatment and Screening of Tea Vareties

The fresh leaves of *C. sinensis* ‘FudingDabai’, *C. sinensis* ‘Mabianlv No. 1’, *C. sinensis* ‘Wuniuzao’ and *C. sinensis* ‘Fuxuan No. 9’ were filled into vacuum bags at 200 g·bag^−1^ and then vacuumed at room temperature. The vacuum degree was less than 0.99 MPa. Then, vacuumed treatment was applied for 0 h, 2 h, 4 h, 6 h, 8 h, and 10 h, separately. Each treatment was repeated three times.

The GABA content in fresh leaves treated with different vacuum times was determined, and the tea varieties in the treatment with higher GABA content were selected as raw materials for the subsequent experiments.

### 2.3. Preparation of Pickled Tea

As shown in Figure S1, the fresh leaves of *C. sinensis* ‘Mabianlv No. 1’ were spread on the green storage tank for 2 h to dissipate moisture. Then, the leaves were packed into vacuum bags with 2000 g per bag and vacuumed. Vacuum treatment times were 0 h, 4 h and 8 h (vacuum degree ≤ 0.99 MPa), respectively. Following this, the leaves were fixed by microwave for 120 s and then rolled for 20 min. The twisted leaves were subdivided into “baked” and “not baked” groups. Those in the “baked” group were dried in an oven at 85 °C for 20 min, whereas those in the “not baked” group were subjected to room temperature for 20 min. The specifical treatments are depicted in Table S1. Afterwards, the tea leaves were pressed into a clean glass bottle very tightly and fully by hammering. The bottles were completely sealed with a cap, divided into different groups randomly, and placed into a closed fermenter at 37 °C for 0 d, 10 d, 20 d, 30 d and 40 d, separately. Finally, the fermented leaves were dried directly at 80 °C. All procedures were repeated three times.

### 2.4. Measurement of Major Chemical Components

The determination of GABA content was performed according to the method described by Wu et al. [18]. The contents of total free amino acids (TFAA), total polyphenols and total acids were determined using spectrophotometry (UV-759, Qingdao Juchuang, Qingdao, China), as previously described [19,20,21]. Epicatechin (Epicatechin, EC), gallate catechin (gallate catechin, GC), epigallocatechin gallate (epigallocatechin gallate, EGC), gallocatechin gallate (gallocatechin gallate, GCG), catechin gallate (catechin gallate, CG), epigallocatechin (epigallocatechin, EGC), catechin (catechin, C) and caffeine (caffeine, CAF) contents of the extracts were determined using HPLC as previously described [22]. Briefly, the catechins in pickled tea was extracted in a 70% aqueous methanol solution at 70 °C and filtered through a 0.22 μm Millipore filter. Then, the samples were analyzed using a 1260 infinity II HPLC system (Agilent Technologies Co., Ltd., Palo Alto, CA, USA) equipped with an ultraviolet detector. Chromatographic separation was performed on a 1260 infinity II liquid chromatography C18 column (250 mm × 4.6 mm, 5 μm)—phase A is a 0.2% glacial acetic acid solution and phase B is acetonitrile, with a flow rate of 1.0 mL·min^-1^ at 35 °C. The optical density was determined at 278 nm.

Further, the water content and water extract content were determined according to GB/T 8304-2013 [23] and GB/T 8305-2013 [24], separately.

### 2.5. Sensory Evaluation

The sensory evaluation of pickled tea was carried out by five professionals (majoring in tea science and qualified as tea part) according to the method described in DB5331/T 39-2023 [25], with slight modifications. Specifically, 3 g of pickled tea was weighed accurately and brewed with 150 mL of boiling water for 5 min. Then, the infusion was filtered out. The color and taste of the infusion, as well as the aroma of infused tea, was evaluated. Password review was adopted, and all sensory assessors did not know any information about the samples. The criteria of the sensory evaluation are shown in Table 1. Scoring is based on a percentage system, in which the weights of appearance, aroma, taste, infusion color and infused leaf are 25%, 25%, 30%, 10% and 5%, respectively.

### 2.6. Statistical Analysis

All experiments were repeated three times. Data are expressed as mean ± SD. Statistical analyses were performed using SPSS (IBM Corp, v23, Armonk, NY, USA). One-way ANOVA with Duncan multiple comparisons tests was used. A value of *p* < 0.05 indicated statistical significance.

## 3. Results

### 3.1. The Biochemical Components of Different Tea Varieties Treated with Different Vacuum Times

**Figure 1:**
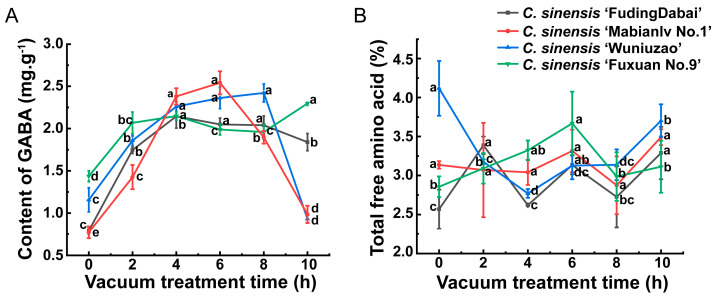
Content of GABA (**A**) and total free amino acid (**B**) of different tea cultivars treated with different vacuum times. Different lowercase letters on the same line indicate statistical significance (*p* < 0.05).

As shown in Figure 1A, the GABA content in *C. sinensis* ‘FudingDabai’, *C. sinensis* ‘Mabianlv No.1’ and *C. sinensis* ‘Wuniuzao’ tended to increase and then gradually decrease with the extension of the vacuum treatment time. The highest level of GABA in *C. sinensis* ‘FudingDabai’, *C. sinensis* ‘Mabianlv No.1’, *C. sinensis* ‘Wuniuzao’ and *C. sinensis* ‘Fuxuan No. 9’ were 2.15 mg·g^−1^, 2.54 mg·g^−1^, 2.42 mg·g^−1^ and 2.30 mg·g^−1^, respectively. These appeared at 4 h, 6 h, 8 h and 10 h of vacuum treatment, separately. In addition, the maximum GABA contents were higher than the standard of 1.5 mg·g^−1^ in GABA-rich tea. As depicted in Figure 1B, the TFAAs of *C. sinensis* ‘FudingDabai’ were the highest at 3.39% for 2 h of vacuum treatment and the lowest at 2.57% without vacuum treatment. Similarly, it was the highest at 3.67% for *C. sinensis* ‘Fuxuan No. 9’, treated for 6 h under vacuum, and lowest at 2.86% without vacuum treatment. In contrast, the TFAA content of *C. sinensis* ‘Wuniuzao’ was highest at 4.12% without vacuum treatment and lowest at 2.77% with 4 h of vacuum treatment. Furthermore, the highest TFAA in *C. sinensis* ‘Mabianlv No. 1’ was observed in the samples vacuumed for 10 h (3.49%), and the lowest was shown in those treated with 8 h of vacuum (2.88%). After vacuum treatment, both the maximum values of GABA and TFAAs appeared in *C. sinensis* ‘Mabianlv No.1’; therefore, *C. sinensis* ‘Mabianlv No.1’ was selected as the raw material for the subsequent experiment.

### 3.2. Major Chemical Components in Different Samples

**Figure 2:**
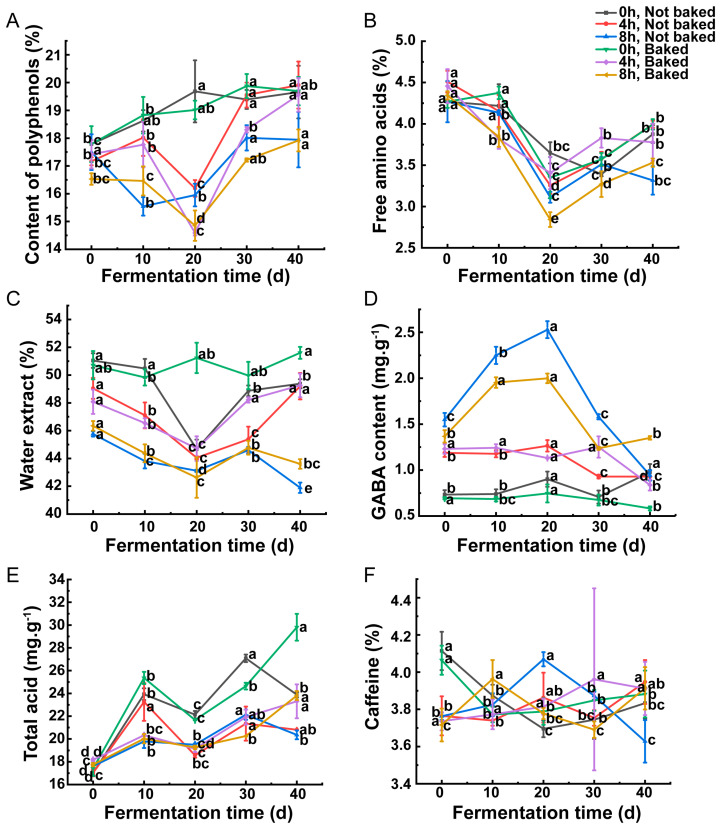
Major chemical components in different samples. Content of total polyphenol content (**A**), total free amino acid (**B**), water extract (**C**), GABA (**D**), total acids (**E**), and caffeine (**F**) in different samples. Different lowercase letters on the same line indicates statistical significance (*p* < 0.05). “0 h/4 h/8 h, not baked” represents the treatment combination of vacuum treatment of fresh leaves for 0 h/4 h/8 h and spreading the leaves for 20 min at room temperature before fermentation, “0 h/4 h/8 h, baked” represents the treatment combination of vacuum treatment of fresh leaves for 0 h/4 h/8 h and drying the leaves in an oven at 85 °C for 20 min before fermentation.

The polyphenol content of the pickled teas made from *C. sinensis* ‘Mabianlv No. 1’ after 8 h of vacuum treatment was significantly lower than those of the non-vacuum treatment group when all other conditions were controlled. In the “baked” treatment group, the tea polyphenol content showed a gradual decrease with the extension of the vacuum treatment time (Figure 2A, Table S2). The total amount of FAAs showed a tendency of decreasing slowly and then increasing gradually with the extension of fermentation time. The highest TFAA amount was 4.51% in the sample with 4 h of vacuum treatment, “not baked” rolled leaves and 0 d of fermentation. The TFAA content of the samples with 8 h of vacuum treatment, “baked” rolled leaves and 20 d of fermentation was the lowest, which was 2.84% (Figure 2B, Table S2). As shown in Figure 2C, with the prolongation of vacuum treatment time, the water extract content trended to decrease. The highest water extract content of 51.2% was observed in the samples treated with 0 h of vacuum, “baked” rolled leaves and fermentation for 20 d. In contrast, the lowest water extract content of 41.89% was detected in the samples treated with vacuum for 8 h, “not baked” rolled leaves and 40 d of fermentation (Figure 2C, Table S2). Except for the treatments of “not baked”, 40 d of fermentation, and “baked”, 30 d of fermentation, the GABA contents tended to increase gradually with the extension of fermentation time (Figure 2D, Table S2). Whether the rolled leaves were baked or not, the highest GABA content was found in the samples treated with vacuum for 8 h and fermented for 20 d, with 2.00 mg·g^−1^ and 2.53 mg·g^−1^, respectively. Among all these samples, the pickled tea presents no vacuum treatment, “baked” and fermented for 40 d, and showed the lowest GABA content of 0.58 mg·g^−1^ (Figure 2D, Table S2). In the “not baked” groups, the total acid showed a trend of increasing and then decreasing with the extension of the fermentation time, and the maximum value of the majority of the samples were observed at 30 d of fermentation. While in the “baked-treatment” groups, the total acid content increased gradually with the extension of fermentation (Figure 2E, Table S2). Among the samples, the highest total acid content of 29.81 mg·g^−1^ was observed in the samples vacuumed for 0 h, “baked” and fermented for 40 d. However, the lowest total acid content of 17.01 mg·g^−1^ was found in the samples vacuumed for 0 h, “baked” and fermented for 0 d (Figure 2E, Table S2). This indicates that fermentation is the main stage for acid formation. As illustrated in Figure 2F and Table S2, the treatments involved in the present study hardly affected the caffeine content, which was stabilized in the range of 3.63% to 4.11%. The lowest caffeine content (3.63%) was observed in the treatment of vacuum for 8 h, without baking and fermented for 40 d. The highest caffeine content was found in those vacuumed for 0 h, without baking and fermented for 0 d.

With the extension of fermentation time, the content of EGCG, ECG and CG showed a decreasing trend, while EGC tended to increase gradually (Table 2). Among all the samples, the highest EGCG content of 11.12% was detected in the sample with 0 h of vacuum treatment, 0 d of fermentation and “not baked” (Sample 1). The lowest EGCG content of 1.85% was obtained by 0 h of vacuum treatment, “not baked” twisted leaves and 30 d of fermentation (Sample 10). In contrast, the highest EGC content of 8.27% was found in the samples with 0 h of vacuum, “not baked” and fermented for 30 d (Sample 10). The lowest EGC content of 2.30% was observed in those with 0 h of vacuum, “not baked” and 0 d of fermentation (Sample 30) (Table 2 and Table S1). Consistent with the total acid content, fermentation time is a major factor contributing to the changes in catechins.

### 3.3. Sensory Evaluation of Different Samples

The results of sensory evaluation showed that with the prolongation of the vacuum treatment time, the infused leaf changed from yellowish green to reddish brown, the taste turned from slightly sour to sour with sweet and mellow, the color of the infusion transformed from light yellowish green to golden yellow and the aroma changed from sour to sour with a fruity and sweet aroma. The infused leaves of the samples fermented for 40 d showed a mud-slippery appearance, and the taste was thick, whereas the color of the infusion was still clear without turbidity. Most of the samples fermented for 20–30 d were characterized by an acidic aroma, and when the infused leaf was complete, the infusion was bright and the quality was the best. However, if the fermentation time continues to be extended, an irregular odor was produced and the quality declined (Table 3 and Table S1).

## 4. Discussion

In the present study, we investigated and optimized the production of GABA pickled tea. Consistent with previous studies [11,12,13,14,15], the content of GABA changed with the external environment, such as oxygen and air pressure. However, we also found that the combination of 8 h of vacuum treatment, 20 min of rolling after microwave fixing, 20 min of spreading and 20 d of anaerobic fermentation at room temperature possess the highest GABA content, as well as a better sensory evaluation. 

GABA content varies with different cultivars [17], and the result of this study also verified this. Except for *C. sinensis* ‘Fuxuan No.9’, the GABA content in the fresh leaves of the other three tea cultivars showed a tendency to first increase and then decrease with the extension of vacuum treatment time. This is in agreement with a previous study [26] in which the GABA content increased first and then decreased, and the highest GABA content was observed in fresh leaves vacuumed for 8 h. In addition, among all treatments, the fresh leaves of *C. sinensis* ‘Mabianlv No. 1’ treated with vacuum for 6 h possess the highest GABA level, as the present study mainly focused on the enrichment of GABA. Therefore, *C. sinensis* ‘Mabianlv No. 1’was selected as the raw material for the subsequent experiments. The differences in the total amount of FAAs of different tea varieties showed that both the vacuum time and tea varieties affect the content of TFAAs significantly, which may be related to factors such as the initial TFAA in fresh leaves and the stress-resistance of fresh leaves.

Under the same treatment conditions, the content of tea polyphenols in fresh leaves after 8 h of vacuuming was lower than that without vacuuming. This may be attributed to the fact that the vacuum treatment damaged the fresh leaves, which leads to the secretion of polyphenol oxidase, thus oxidizing the tea polyphenols. With the extension of vacuum treatment time, the oxidation degree of tea polyphenols increased and the tea polyphenol content declined. With the prolongation of fermentation, the total amount of FAAs showed a trend of decline followed by gradual recovery. This may be caused by the microorganisms produced in the fermentation stage. In the early period, amino acids were consumed by microorganisms, whereas in the middle and late stages, proteins were decomposed into amino acids or polypeptides by microorganisms, and therefore the content of amino acids increased. The water extract content of the different samples in the present study ranged from 41.89% to 51.23%, which is consistent with the results of a previous study [27]. The content of water extracts decreased with the prolongation of the vacuum treatment time. This might be attributed to the fact that the vacuum treatment led to the combination of soluble substances with macromolecules and transformed them into insoluble substances. Nevertheless, the water extract content decreased and then increased with the extension of the fermentation time. This is because the soluble substances were consumed by microorganisms in the early stage. While in the middle and late stages, the microorganisms decomposed the insoluble macromolecules into small soluble substances. Similarly, the fermentation time affected the GABA content, which may be attributed to the high decomposability of GABA. GABA produced during the pre-fermentation period may be degraded due to temperature changes during fermentation. The content of total acid content in different samples showed that the condition of being “baked” did not affect its content. However, with the extension of fermentation time, the total acid content gradually increased, which is consistent with the results of Wei et al. [28]. This also indicates that fermentation is the main process for the formation of the “sour flavor” in pickled tea. Furthermore, it was found that the maximum value of total acid appeared earlier in the samples of the “baked” treatment group than that of the “not baked” treatment group, which was due to the fact that being “baked” reduced the moisture of the leaves, and this slowed down the fermentation process.

As the fermentation proceeded, the content of bitter and astringent ester catechins such as EGCG, ECG and CG decreased gradually, while the content of milder tasting non-ester catechins such as EGC tended to increase. This result is in agreement with previous studies [3,29]. Meanwhile, this coincided with the results of sensory evaluation, indicating that proper fermentation is helpful to reduce the bitterness and astringency of summer and autumn teas. The caffeine content of the different samples ranged from 3.63% to 4.11%, which is consistent with the result observed by Liang et al. [27]. Meanwhile, the different treatments in this study had little effect on the caffeine content, which is owing to the stable structure of caffeine, and the content of caffeine remained basically unchanged with little change in the temperature.

With the extension of vacuum treatment time, the aroma, taste, color and infused leaf underwent transformation and eventually formed the unique quality characteristics of pickled tea. This is attributed to the damage to the leaves caused by vacuum treatment, which promotes the transformation of bitter and astringent substances and the formation of fruity and floral substances. Therefore, moderate damage to the leaves is conducive to the transformation of pickled tea quality. As fermentation proceeds, the infusion presented a mellow taste, which is because during fermentation, the cellulose and other macromolecules in the tea leaves are decomposed by microorganisms and transformed into soluble small molecules. However, a long fermentation time will lead to mud-slip on the infused leaf and aggravate the taste. Therefore, from the results of this study, fermentation should be terminated at 20–30 d to prevent deterioration.

## 5. Conclusions

In this study, we firstly found that *C. sinensis* ‘Mabianlv No.1’ was the best cultivar for pickled tea production by the vacuum treatment of the fresh leaves of different tea varieties. The highest GABA content of 2.53 mg·g^−1^ was obtained from the fresh leaves of *C. sinensis* ‘Mabianlv No.1’ after 8 h of vacuum treatment with microwave fixing, 20 min of rolling, 20 min of spreading and 20 d of anaerobic fermentation at room temperature. Sensory evaluation showed that the pickled tea had a strong and lasting sour aroma, the taste is thick, refreshing, back to sweet, slightly acidic produce fluid and strong. The content of tea polyphenols, TFAAs, water extract, total acids and caffeine of the pickled tea samples obtained by this process was 15.95%, 3.11%, 43.11%, 19.50 mg·g^−1^ and 4.07%, respectively. These results indicated that this is the best process for the production of pickled tea.

## Figures and Tables

**Table 1:** Criteria for the sensory evaluation of pickled tea.

Appearance	Aroma	Taste	Color	Infused Leaf	Score
Plump and tender, tight, yellow and brown moist bud, even and clean	Strong sour aroma, lasting	Concentrated mellow sweet, slightly acid, produce fluid	Bright orange yellow	Tender buds appear	90~100
Plump and tight, brown and green, moist, even and clean	Strong sour aroma	Concentrated mellow back to sweet, slightly acid, produce fluid	Yellow, thick and bright	Tender, with bud	85~90
Tight, brown and green, moist, even and clean, with tender stem	Strong and pure sour aroma	Mellow and rich, back to sweet, slightly acid, produce fluid	Yellow, thick, fairly bright	Tender and even	80~85
Firm, brown, moist, even, with less stem	Sour aroma, fairly strong	Thick back to sweet, slightly acid, produce fluid	Yellow, less bright	stout and strong	75~80
Fairly firm, brown, less moist, fairly even, with stem	Pure sour aroma	Mellow and back to sweet, slightly acid, produce fluid	yellow, slightly turbid	thickset	70~75
Coarse loose, brown, slightly uneven, not even, with stalk	Sour aroma, plain	Pure back to sweet, slightly acid, produce fluid	yellow, turbid	coarse	60~70

**Table 2:** The content of catechins in different samples.

Sample Number	GC/%	EGC/%	C/%	EC/%	EGCG/%	GCG/%	ECG/%	CG/%
1	1.06 ± 0.46 ^c^	4.05 ± 0.41 ^lmn^	0.12 ± 0.00 ^cde^	2.41 ± 0.03 ^efghij^	11.12 ± 0.71 ^a^	0.31 ± 0.11 ^abc^	2.25 ± 0.11 ^a^	0.71 ± 0.02 ^a^
2	3.05 ± 2.24 ^ab^	4.03 ± 0.03 ^lmn^	0.15 ± 0.01 ^cde^	2.14 ± 0.05 ^ijklm^	9.66 ± 0.25 ^b^	/	1.97 ± 0.06 ^b^	0.59 ± 0.01 ^efgh^
3	0.73 ± 0.00 ^c^	3.03 ± 0.02 ^q^	0.14 ± 0.00 ^de^	1.77 ± 0.02 ^nop^	8.06 ± 0.06 ^cd^	/	1.88 ± 0.03 ^bc^	0.65 ± 0.02 ^bcd^
4	1.14 ± 0.05 ^c^	7.07 ± 0.16 ^c^	0.16 ± 0.00 ^cde^	3.30 ± 0.07 ^ab^	3.44 ± 0.20 ^p^	0.33 ± 0.03 ^ab^	0.51 ± 0.03 ^m^	0.55 ± 0.01 ^ghijk^
5	1.24 ± 0.02 ^c^	4.24 ± 0.05 ^klm^	0.18 ± 0.00 ^cde^	2.40 ± 0.05 ^fghij^	7.26 ± 0.31 ^efg^	0.261 ± 0.02 ^cde^	1.41 ± 0.06 ^efg^	0.58 ± 0.01 ^efgh^
6	1.04 ± 0.03 ^c^	3.11 ± 0.31 ^pq^	0.17 ± 0.00 ^cde^	1.77 ± 0.08 ^nop^	7.44 ± 0.56 ^ef^	0.26 ± 0.01 ^bcde^	1.59 ± 0.13 ^d^	0.61 ± 0.02 ^defg^
7	1.02 ± 0.12 ^c^	5.82 ± 0.02 ^ef^	0.16 ± 0.03 ^cde^	2.94 ± 0.01 ^cd^	5.26 ± 0.13 ^klm^	0.29 ± 0.05 ^abcde^	0.87 ± 0.02 ^jk^	0.54 ± 0.01 ^hijk^
8	1.05 ± 0.04 ^c^	3.71 ± 0.09 ^no^	0.16 ± 0.03 ^cde^	1.34 ± 0.83 ^qr^	7.68 ± 0.30 ^cde^	0.27 ± 0.00 ^bcde^	1.57 ± 0.06 ^d^	0.58 ± 0.01 ^efgh^
9	1.15 ± 0.01 ^c^	4.52 ± 0.04 ^jk^	0.18 ± 0.03 ^cde^	2.21 ± 0.04 ^hijkl^	5.19 ± 0.17 ^lm^	0.22 ± 0.01 ^e^	1.11 ± 0.04 ^hi^	0.63 ± 0.01 ^cde^
10	1.19 ± 0.03 ^c^	8.27 ± 0.25 ^a^	0.36 ± 0.05 ^a^	3.33 ± 0.10 ^a^	1.85 ± 0.05 ^q^	0.31 ± 0.00 ^abc^	0.29 ± 0.01 ^n^	0.51 ± 0.02 ^ijk^
11	1.46 ± 0.02 ^bc^	5.29 ± 0.12 ^gh^	0.21 ± 0.06 ^c^	2.25 ± 0.06 ^hijk^	4.81 ± 0.13 ^mn^	0.29 ± 0.00 ^bcde^	1.03 ± 0.03 ^i^	0.55 ± 0.01 ^ghijk^
12	1.29 ± 0.01 ^c^	5.55 ± 0.05 ^fg^	0.28 ± 0.05 ^b^	2.07 ± 0.02 ^jklmn^	2.23 ± 0.06 ^q^	0.24 ± 0.01 ^de^	0.49 ± 0.01 ^m^	0.62 ± 0.00 ^cdef^
13	1.51 ± 0.36 ^bc^	5.60 ± 0.15 ^fg^	0.16 ± 0.06 ^cde^	2.49 ± 0.21 ^efghi^	5.27 ± 0.21 ^klm^	0.23 ± 0.01 ^e^	0.81 ± 0.03 ^jkl^	0.50 ± 0.13 ^k^
14	1.72 ± 0.02 ^bc^	4.82 ± 0.15 ^ij^	0.15 ± 0.00 ^cde^	2.21 ± 0.10 ^hijkl^	7.01 ± 0.08 ^fgh^	0.24 ± 0.02 ^e^	1.31 ± 0.02 ^g^	0.57 ± 0.01 ^efgh^
15	1.40 ± 0.04 ^bc^	4.45 ± 0.10 ^jk^	0.14 ± 0.00 ^de^	1.89 ± 0.04 ^lmnop^	3.54 ± 0.05 ^p^	0.23 ± 0.03 ^e^	0.75 ± 0.01 ^l^	0.51 ± 0.01 ^jk^
16	1.03 ± 0.40 ^c^	4.15 ± 0.21 ^klm^	0.15 ± 0.00 ^cde^	2.27 ± 0.06 ^hijk^	10.82 ± 0.57 ^a^	0.30 ± 0.10 ^abcd^	2.20 ± 0.14 ^a^	0.70 ± 0.02 ^ab^
17	4.12 ± 3.36 ^a^	3.91 ± 0.05 ^mn^	0.16 ± 0.00 ^cde^	2.18 ± 0.03 ^hijklm^	9.38 ± 0.23 ^b^	/	1.94 ± 0.06 ^bc^	0.59 ± 0.10 ^efgh^
18	2.34 ± 2.89 ^bc^	3.05 ± 0.02 ^q^	0.15 ± 0.00 ^cde^	1.85 ± 0.05 ^mnop^	8.08 ± 0.29 ^c^	0.27 ± 0.01 ^bcde^	1.84 ± 0.08 ^c^	0.66 ± 0.02 ^bc^
19	0.98 ± 0.05 ^c^	6.45 ± 0.06 ^d^	0.18 ± 0.03 ^cd^	3.01 ± 0.04 ^bc^	4.42 ± 0.14 ^no^	0.35 ± 0.01 ^a^	0.79 ± 0.03 ^kl^	0.58 ± 0.01 ^efgh^
20	0.87 ± 0.02 ^c^	4.35 ± 0.12 ^kl^	0.16 ± 0.00 ^cde^	2.38 ± 0.06 ^fghij^	7.63 ± 0.23 ^cde^	0.24 ± 0.01 ^de^	1.51 ± 0.05 ^de^	0.59 ± 0.01 ^efgh^
21	0.86 ± 0.10 ^c^	3.27 ± 0.09 ^pq^	0.18 ± 0.03 ^cd^	1.94 ± 0.05 ^klmno^	7.54 ± 0.22 ^de^	0.23 ± 0.01 ^e^	1.59 ± 0.05 ^d^	0.63 ± 0.02 ^cde^
22	1.02 ± 0.01 ^c^	5.56 ± 0.09 ^fg^	0.19 ± 0.00 ^cd^	2.70 ± 0.06 ^cdef^	6.23 ± 0.27 ^ij^	0.23 ± 0.00 ^e^	1.17 ± 0.06 ^h^	0.59 ± 0.01 ^efgh^
23	1.10 ± 0.05 ^c^	5.08 ± 0.13 ^hi^	0.22 ± 0.01 ^c^	2.52 ± 0.07 ^efgh^	7.00 ± 0.27 ^fgh^	0.27 ± 0.01 ^bcde^	1.38 ± 0.06 ^fg^	0.57 ± 0.01 ^efghi^
24	0.86 ± 0.02 ^c^	2.96 ± 0.03 ^q^	0.18 ± 0.07 ^cd^	1.60 ± 0.04 ^pq^	6.84 ± 0.25 ^gh^	0.25 ± 0.01 ^cde^	1.46 ± 0.07 ^ef^	0.60 ± 0.01 ^defgh^
25	0.93 ± 0.12 ^c^	5.37 ± 0.04 ^gh^	0.18 ± 0.07 ^cd^	2.30 ± 0.07 ^ghij^	4.93 ± 0.17 ^m^	0.25 ± 0.01 ^cde^	0.91 ± 0.04 ^j^	0.57 ± 0.02 ^efgh^
26	1.23 ± 0.15 ^c^	6.08 ± 0.83 ^de^	0.20 ± 0.08 ^cd^	2.74 ± 0.29 ^cde^	5.75 ± 0.56 ^jk^	0.25 ± 0.02 ^cde^	1.12 ± 0.11 ^hi^	0.60 ± 0.07 ^defgh^
27	0.93 ± 0.01 ^c^	3.47 ± 0.01 ^op^	0.19 ± 0.07 ^cd^	1.73 ± 0.06 ^op^	5.53 ± 0.31 ^kl^	0.23 ± 0.04 ^e^	1.16 ± 0.08 ^h^	0.56 ± 0.01 ^fghi^
28	1.41 ± 0.12 ^bc^	7.69 ± 0.31 ^b^	0.15 ± 0.01 ^cde^	3.41 ± 0.31 ^a^	3.06 ± 0.10 ^p^	0.31 ± 0.00 ^abc^	0.53 ± 0.02 ^m^	0.55 ± 0.02 ^ghijk^
29	1.40 ± 0.06 ^bc^	6.31 ± 0.32 ^d^	0.15 ± 0.01 ^cde^	2.64 ± 0.11 ^defg^	4.23 ± 0.15 ^o^	0.23 ± 0.00 ^e^	0.81 ± 0.02 ^jkl^	0.56 ± 0.01 ^fghij^
30	1.00 ± 0.03 ^c^	2.30 ± 0.07 ^r^	0.11 ± 0.01 ^e^	1.16 ± 0.03 ^r^	6.56 ± 0.29 ^hi^	0.27 ± 0.05 ^bcde^	1.36 ± 0.05 ^fg^	/

Note: “/” stands for “Not detected”. Different superscript letters in the same column indicates statistical significance (*p* < 0.05).

**Table 3:** Sensory evaluation results of different samples.

Sample Number	Appearance	Aroma	Taste	Color	Infused Leaf	Score
4	Coarse loose yellow film, yellow-brown, even	Sour aroma, pure	Thick, refreshing, back to sweet	Light yellow-green, clear, bright	Soft, bright, complete, yellow-green	74.77 ± 0.71 ^gh^
5	Coarse loose yellow film, yellow-brown, even and clean	Sour aroma, pure	Thick, refreshing, back to sweet	Pale yellow, clear and bright	Soft, bright, complete, yellow-green	74.82 ± 1.28 ^gh^
6	Slightly firm, yellow-brown, even	Sour aroma, pure	Thick, refreshing, back to sweet	Light yellow-green, clear, bright	Soft, bright, complete, yellow-green	77.30 ± 0.31 ^e^
7	Slightly firm, yellow-brown, even	Sour aroma, pure	Thick, refreshing, back to sweet	Light yellow-green, clear, bright	Soft, bright, complete, yellow-green	76.70 ± 0.61 ^ef^
8	Coarse loose yellow film, yellow-brown, even	Sour aroma, strong	Thick, refreshing, back to sweet	Pale yellow, slightly muddy, bright	Soft, bright, complete, yellow-green	78.12 ± 0.60 ^e^
9	Coarse loose yellow film, yellow-brown even	Sour aroma, strong and lasting	Thick, refreshing, back to sweet, slightly acid produce fluid	Light apricot-yellow, slightly muddy, bright	Soft, bright, complete, red	82.98 ± 0.24 ^b^
10	Slightly firm, yellow-brown, even and clean	Sour aroma, strong and lasting	Thick, refreshing, back to sweet, sour	Light yellow-green, clear, bright	Soft, bright, complete, yellow-green	84.42 ± 0.83 ^a^
11	Slightly firm, yellow-brown, even	Sour aroma, strong and lasting	Thick, refreshing, back to sweet, sour	Yellow-green, clear and bright	Soft, bright, complete, yellow-green	82.65 ± 1.29 ^b^
12	Coarse loose yellow film, yellow-brown, even and clean	Sour aroma, strong and lasting	Thick, refreshing, back to sweet, slightly sweet	Golden, clear and bright	Soft, bright, complete, red	85.08 ± 1.08 ^a^
13	Slightly firm, yellow-brown, even	Rich sour aroma, pungent smell	Thick, refreshing, back to sweet, sour	Pale yellow, clear and bright	Soft, bright, complete, yellow-green	74.03 ± 0.75 ^h^
14	Slightly firm, yellow-brown, even and clean	Sour aroma, strong and lasting	Thick, refreshing, back to sweet, slightly acid produce fluid	Pale yellow, clear and bright	Soft, bright, complete, yellow-green, slightly slimy	85.32 ± 1.23 ^a^
15	Coarse loose yellow film, yellow-brown, even and clean	Sour aroma, pure	Thick, refreshing, back to sweet, slightly acid produce fluid	Golden, clear and bright	Soft, bright, complete, yellow-green, slightly slimy	80.25 ± 0.61 ^cd^
19	Slightly firm, yellow-brown, even	Sour aroma, pure	Thick, refreshing, back to sweet	Pale yellow, clear and bright	Soft, bright, complete, yellow-green	77.43 ± 0.31 ^e^
20	Coarse loose yellow film, yellow-brown, even	Sour aroma, pure	Thick, refreshing, back to sweet	Light yellow-green, clear, bright	Soft, bright, complete, red	75.00 ± 0.69 ^gh^
21	Coarse loose yellow film, yellow-brown, even	Sour aroma, pure	Thick, refreshing, back to sweet, sweet alcohol	Pale yellow, clear and bright	Soft, bright, complete, red	79.93 ± 0.46 ^d^
22	Coarse loose yellow film, yellow-brown, even	Sour aroma, pure	Thick, refreshing, back to sweet	Light yellow green, clear, bright	Soft, bright, complete, yellow-green, slightly slimy	74.80 ± 0.53 ^gh^
23	Coarse loose yellow film, yellow-brown, even	Sour aroma, pure	Thick, refreshing, back sweet	Yellow-green, clear and bright	Soft, bright, complete, yellow-green	74.80 ± 0.63 ^gh^
24	Coarse loose yellow film, yellow-brown even	Pure sour flavor	Thick, refreshing, back to sweet, slightly acid produce fluid	Apricot-yellow, clear and bright	Soft, bright, complete, red	79.70 ± 0.13 ^d^
25	Slightly firm, yellow-brown, even	Pure sour flavor	Thick, refreshing, back sweet, sour	Light yellow-green, clear, bright	Soft, bright, complete, yellow-green, slightly slimy	75.28 ± 0.86 ^fgh^
26	Slightly firm, yellow-brown, even and clean	Rich sour flavor	Thick, refreshing, back to sweet, slightly acid produce fluid	Pale yellow, clear and bright	Soft, bright, complete, yellow-green, slightly slimy	85.98 ± 0.78 ^a^
27	Coarse loose yellow film, yellow-brown even and clean	Rich sour flavor	Thick, refreshing, back to sweet, slightly acid produce fluid	Golden, clear and bright	Soft, bright, complete, red	85.35 ± 0.66 ^a^
28	Slightly firm, yellow-brown, even	Rich sour flavor, Pungent smell	Thick, refreshing, back sweet, acidic	Pale yellow, clear and bright	Soft, bright, complete, yellow-green, slightly slimy	75.78 ± 1.23 ^fg^
29	Slightly firm, yellow-brown, even and clean	Pure sour flavor	Thick, refreshing, back to sweet, slightly acid produce fluid	Pale yellow, clear and bright	Soft, bright, complete, yellow-green, slightly slimy	81.60 ± 1.30 ^bc^
30	Coarse loose yellow film, yellow-brown, even and clean	Rich sour flavor	Thick, refreshing, back to sweet, acid, produce fluid	Golden, clear and bright	Soft, bright, complete, yellow-green, slightly slimy	82.28 ± 1.53 ^b^

Note: Samples numbered 1–3 and 16–18 were not fermented and were not subjected to sensory evaluation. Different superscript letters in the same column indicates statistical significance (*p* < 0.05).

## Data Availability

The original contributions presented in the study are included in the article/Supplementary Materials, further inquiries can be directed to the corresponding author.

## Supplementary Materials

The following supporting information can be downloaded at: https://www.mdpi.com/article/10.3390/foods13142287/s1. Figure S1. Diagram of pickled tea processing. Figure S2. The HPLC chromatogram of the randomly selected sample. Table S1. Information for different pickled tea samples. Table S2. Original data of Figure 2.
