# Supplementary material for: Process Optimization and Quality Components Analysis of γ-Aminobutyric Acid Pickled Tea
Source: Foods. 2024 Jul 20;13(14):2287. doi: 10.3390/foods13142287 (PMC11276515; doi:10.3390/foods13142287)
Supplement: Supplementary file 1 [file foods-13-02287-s001.zip › foods-3088504-Supplementary materials_revised R2.pdf]

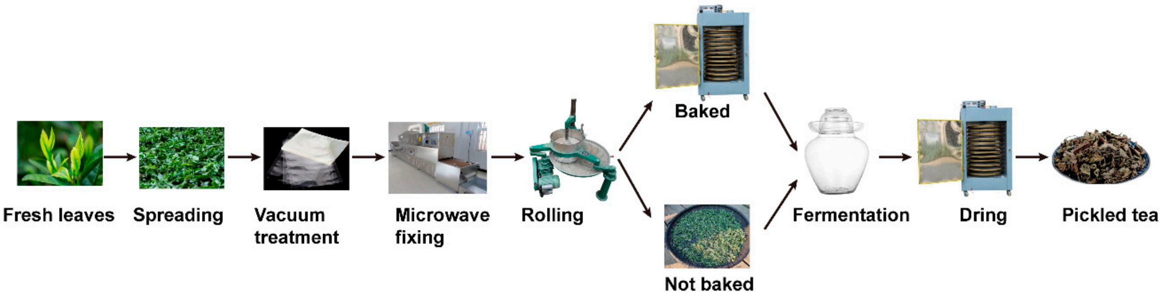

Figure S1. Diagram of pickled tea processing.

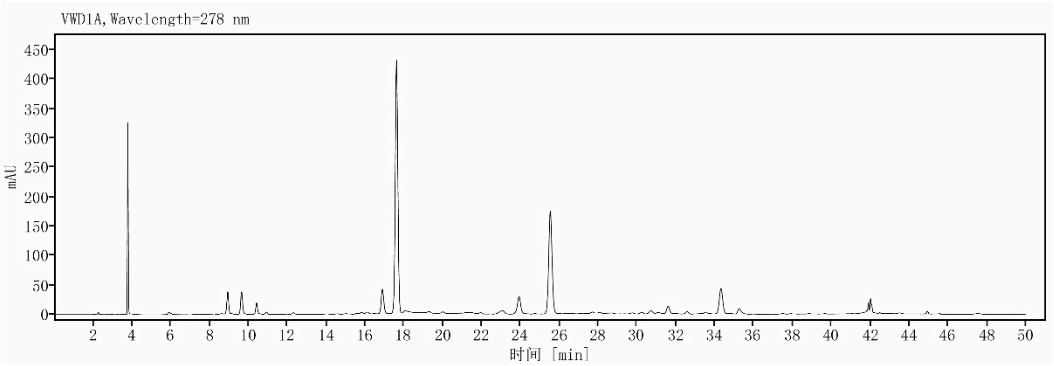

Signal VWD1A, Wavelength=278 nm

RT(min)	Peak width (min)	Peak area	Height	Peak area (%)
12.280	0.61	28.71	2.78	0.38
16.869	0.65	392.93	41.80	5.15
17.596	0.60	4011.80	431.32	52.61
19.256	0.45	29.42	2.71	0.39
23.902	1.04	381.09	30.21	5.00
25.514	1.05	2105.25	174.92	27.61
28.070	0.35	16.78	1.76	0.22
34.301	0.69	540.87	43.47	7.09
35.239	0.76	118.49	9.29	1.55
	Total	7625.35		

Figure S2. The HPLC chromatogram of the randomly selected sample.

Table S1. Information for different pickled tea samples.

Sample number	Not baked or Baked	Fermentation Time/d	Vacuum Time/h
1	Not baked	0	0
2	Not baked	0	4
3	Not baked	0	8
4	Not baked	10	0
5	Not baked	10	4
6	Not baked	10	8
7	Not baked	20	0
8	Not baked	20	4
9	Not baked	20	8
10	Not baked	30	0
11	Not baked	30	4
12	Not baked	30	8
13	Not baked	40	0
14	Not baked	40	4
15	Not baked	40	8
16	Baked	0	0
17	Baked	0	4
18	Baked	0	8
19	Baked	10	0
20	Baked	10	4
21	Baked	10	8
22	Baked	20	0
23	Baked	20	4
24	Baked	20	8
25	Baked	30	0
26	Baked	30	4
27	Baked	30	8
28	Baked	40	0
29	Baked	40	4
30	Baked	40	8

Table S2 Original data of Figure 2.

Vacuum time (h)	Treatment for rolled leaves	Fermentation time (d)	Polyphenol content (%)	Free amino acid (%)	Water extract (%)	GABA content (mg.g^{-1})	Total acid (mg. g^{-1})	Caffeine (%)
0	Not baked	0	17.79±0.65^{def}	4.27±0.07^{abc}	51.05±0.53^{ab}	0.73±0.05^{kl}	17.01±0.23^{n}	4.11±0.10^{a}
0	Not baked	10	18.63±0.38^{bcd}	4.22±0.26^{bcd}	50.48±0.68^{abc}	0.74±0.05^{kl}	23.93±0.51^{d}	3.87±0.06^{bcdef}
0	Not baked	20	19.68±1.12^{ab}	3.65±0.13^{fghi}	44.67±0.69^{igk}	0.90±0.08^{ij}	22.13±0.33^{ef}	3.70±0.05^{fg}
0	Not baked	30	19.39±0.34^{ab}	3.39±0.03^{ijk}	48.87±0.39^{de}	0.71±0.07^{l}	27.08±0.33^{b}	3.74±0.10^{defg}
0	Not baked	40	19.66±0.98^{ab}	3.88±0.11^{ef}	49.38±0.33^{cde}	0.98±0.09^{i}	23.88±0.24^{d}	3.83±0.08^{cdefg}
0	baked	0	17.79±0.65^{def}	4.27±0.07^{abc}	50.72±1.02^{ab}	0.69±0.01^{l}	17.41±0.66^{mn}	4.06±0.08^{abc}
0	baked	10	18.83±0.66^{abcd}	4.38±0.08^{abc}	49.83±0.58^{bcd}	0.68±0.03^{lm}	25.36±0.54^{c}	3.77±0.04^{defg}
0	baked	20	19.01±0.34^{abc}	3.35±0.25^{jkl}	51.23±1.09^{a}	0.75±0.10^{kl}	21.66±0.03^{fg}	3.79±0.07^{defg}
0	baked	30	19.87±0.43^{a}	3.57±0.08^{ghij}	49.97±0.98^{bcd}	0.67±0.06^{lm}	24.64±0.29^{cd}	3.85±0.06^{bcdefg}
0	baked	40	19.69±0.51^{ab}	3.99±0.06^{de}	51.60±0.43^{a}	0.58±0.02^{m}	29.81±1.18^{a}	3.88±0.13^{bcdef}
4	Not baked	0	17.17±0.14^{fgh}	4.51±0.13^{a}	49.04±0.75^{de}	1.19±0.05^{gh}	17.03±0.31^{n}	3.77±0.11^{defg}
4	Not baked	10	18.02±0.66^{cdef}	4.15±0.20^{cd}	47.10±0.93^{fg}	1.18±0.04^{gh}	23.25±1.66^{de}	3.74±0.03^{defg}
4	Not baked	20	16.19±0.30^{hi}	3.26±0.10^{kl}	44.05±0.11^{kl}	1.26±0.06^{fg}	18.57±0.21^{klm}	3.86±0.13^{bcdefg}
4	Not baked	30	19.55±0.45^{ab}	3.57±0.09^{ghij}	45.39±0.90^{hig}	0.93±0.02^{ij}	21.35±1.50^{fgh}	3.75±0.04^{defg}
4	Not baked	40	19.91±0.85^{a}	4.00±0.04^{de}	49.20±0.95^{de}	0.93±0.00^{ij}	20.81±0.03^{fghi}	3.95±0.12^{abcde}
4	baked	0	17.42±0.51^{efg}	4.46±0.21^{ab}	48.09±0.87^{ef}	1.23±0.05^{gh}	18.17±0.11^{lmn}	3.74±0.05^{defg}
4	baked	10	17.76±0.82^{def}	3.82±0.12^{efg}	46.55±0.35^{gh}	1.24±0.04^{g}	20.33±0.07^{ghij}	3.77±0.08^{defg}
4	baked	20	14.59±0.09^{j}	3.41±0.20^{ijk}	44.71±0.88^{igk}	1.13±0.01^{h}	19.13±0.28^{jkl}	3.81±0.04^{defg}
4	baked	30	18.30±0.10^{cde}	3.83±0.12^{ef}	48.22±0.19^{ef}	1.25±0.12^{fg}	21.92±0.66^{ef}	3.96±0.49^{abcd}
4	baked	40	19.56±0.61^{ab}	3.78±0.22^{efgh}	49.26±0.86^{cde}	0.84±0.06^{jk}	23.30±1.49^{de}	3.91±0.14^{abcdef}
8	Not baked	0	17.50±0.64^{efg}	4.27±0.25^{abc}	45.77±0.19^{hi}	1.55±0.07^{d}	17.61±0.05^{mn}	3.76±0.02^{defg}
8	Not baked	10	15.55±0.33^{ij}	4.14±0.03^{cd}	43.81±0.53^{klm}	2.25±0.09^{b}	19.79±0.58^{ijk}	3.83±0.06^{defg}

8	Not baked	20	15.95 ± 0.41^{i}	3.11 ± 0.07^{l}	43.11 ± 0.08^{lm}	2.53 ± 0.09^{a}	19.50 ± 0.11^{ijkl}	4.07 ± 0.04^{ab}
8	Not baked	30	18.01 ± 0.45^{cdef}	3.51 ± 0.09^{ijk}	44.60 ± 0.24^{igk}	1.58 ± 0.03^{d}	22.14 ± 0.46^{ef}	3.87 ± 0.02^{bcdef}
8	Not baked	40	17.95 ± 1.00^{def}	3.32 ± 0.17^{jkl}	41.89 ± 0.38^{n}	0.94 ± 0.06^{ij}	20.36 ± 0.39^{ghij}	3.63 ± 0.11^{g}
8	baked	0	16.53 ± 0.21^{ghi}	4.35 ± 0.05^{abc}	46.35 ± 0.35^{gh}	1.37 ± 0.07^{e}	17.75 ± 0.15^{mn}	3.72 ± 0.09^{efg}
8	baked	10	16.46 ± 0.52^{ghi}	3.84 ± 0.12^{ef}	44.37 ± 0.65^{gk}	1.95 ± 0.06^{c}	20.02 ± 0.40^{hij}	3.96 ± 0.10^{abcd}
8	baked	20	14.85 ± 0.55^{j}	2.84 ± 0.09^{m}	42.62 ± 1.46^{mn}	2.00 ± 0.05^{c}	19.26 ± 0.16^{jkl}	3.78 ± 0.03^{defg}
8	baked	30	17.21 ± 0.06^{efgh}	3.27 ± 0.16^{kl}	44.78 ± 0.50^{igk}	1.24 ± 0.02^{gh}	20.253 ± 0.01^{ghij}	3.691 ± 0.04^{fg}
8	baked	40	17.92 ± 0.40^{def}	3.53 ± 0.05^{hij}	43.60 ± 0.34^{klm}	1.35 ± 0.02^{ef}	23.81 ± 0.39^{d}	3.91 ± 0.11^{abcdef}

Note: Different superscript letters in the same column and the same color indicates statistical significance ($p < 0.05$).
